# Development and validation of a score for emergency intervention in patients with acute renal colic secondary to ureteric stones

**DOI:** 10.1080/2090598X.2020.1761143

**Published:** 2020-05-19

**Authors:** Abdullatif Al-Terki, Ahmed R. El-Nahas, Usama Abdelhamid, Mohamed A. Al-Ruwaished, Talal Alanzi, Tariq F. Al-Shaiji

**Affiliations:** a Urology Unit, Al-Amiri Hospital, Kuwait City, Kuwait; b Professor of Urology, Urology and Nephrology Center, Mansoura University, Mansoura, Egypt; c Urology Consultant, Al-Amiri Hospital, Gulf Road, Sharq, Kuwait City, Kuwait; d Urology Unit, Farwaniya Hospital, Farwaniyah Governorate, Farwaniyah, Kuwait

**Keywords:** Renal colic, emergency, ureteric stone, stent, score

## Abstract

**Objectives**: To develop and validate a scoring system to assess the need for emergency intervention (EI) in patients with uncomplicated acute renal colic (ARC) due to ureteric stones.

**Patients and methods**: From May 2017 to April 2019, 382 adult patients presented to emergency department with ARC due to ureteral stones diagnosed by non-contrast computed tomography. Patients with solitary kidney, complications secondary to obstruction (intractable vomiting, fever or sepsis), bilateral ureteric stones, Stage ≥3 chronic kidney disease or those who underwent treatment of urolithiasis within the past 6 months were excluded. EI was performed in cases with persistent or recurrent pain despite analgesics. Multivariate analysis was performed for the first 200 patients to detect risk factors for EI. The score was developed from significant factors. Sensitivity and specificity of the ARC score were calculated using receiver operator characteristic (ROC) curve analysis. The data of last 182 patients were used for validation of the score.

**Results**: In the first 200 patients, EI was needed in 119 patients (59.5%) and included ureteric stents in 92, ureteroscopy in 25 and percutaneous nephrostomy in two. Significant factors for EI were stone location (relative risk [RR] 3.34, *P* = 0.026), creatinine level (RR 1.04, *P* < 0.001), leucocyte count (RR 1.69, *P* < 0.001), and stone length (RR 1.85, *P* < 0.001). A score using these four variables was developed. The ARC score sensitivity was 86%, specificity was 80% and the area under the ROC curve was 0.902. Validation of the score showed strong correlation between ARC score and need for EI (*r* = 0.788, *P* < 0.001).

**Conclusions**: The ARC score is a validated, highly sensitive and specific novel score to determine the need for EI in patients with uncomplicated ARC secondary to ureteric stones.

**Abbreviations:** ARC: acute renal colic; AUC: area under the ROC curve; CDR: clinical decision rules; CKD: chronic kidney disease; ED: emergency department; EI: emergency intervention; MET: medical expulsive therapy; NCCT: non-contrast CT; PCNL, percutaneous nephrolithotomy; ROC: receiver operator characteristic; S.T.O.N.E.: stone size (S), tract length (T), obstruction (O), number of involved calyces (N), and essence or stone density (E); SWL: extracorporeal shockwave lithotripsy; URS: ureteroscopy; WBC: white blood cell

## Introduction

The rate of emergency department (ED) visits for urolithiasis is steadily increasing [1,2]. Acute renal colic (ARC) secondary to ureteric stones is the most common presentation among those patients. The primary goal of the ED physician is to relieve this severe pain by different types of analgesics [2]. When pain is controlled, the patient will be referred to the urologist for treatment of their ureteric calculi. Treatment options include medical expulsive therapy (MET), extracorporeal shockwave lithotripsy (SWL) or ureteroscopy (URS) [3].

In some cases, analgesics are not enough because of persistent pain or development of complications secondary to upper tract obstruction. Such patients require emergency intervention (EI) by either renal drainage (using a ureteric stent or a nephrostomy tube) or by undergoing emergency URS. However, the response of patients to analgesics is subjective, as some patients can tolerate severe pain and others may have consequences of upper tract obstruction with little or no pain. Moreover, cessation of pain does not mean that the ureteric stone has passed [4]. Identifying patients who need EI based on objective data is important to help physicians in decision making to avoid repeated ED visits due to recurrent pain or discharging patients from the ED with complications secondary to upper tract obstruction.

The need for urological intervention after discharge from the ED has been investigated in several studies. Papa et al. [5] in 2005 proposed certain criteria for patients who will require intervention within 28 days after discharge from ED, but in 2016, this model failed external validation [6]. In 2015, Yan et al. [7] identified eight significant risk factors for the need of intervention within 90 days of an ED visit.

Because of the lack of validated measurements that define the need for EI for patients presenting to the ED with ARC secondary to ureteric stones, the present study was conducted to develop and validate a scoring system based on objective data for the need of EI in these patients.

## Patients and methods

A prospective observational study design was approved by the Institutional Review Board of the Surgery Department, Al-Amiri Hospital, Kuwait. All adult patients who presented to the ED in our hospital complaining of ARC were tested for eligibility. Initial patients’ evaluation was conducted by the ED physician and included pain severity score (from 1 to 10), medical and surgical history, vital signs, abdominal examination, and ultrasonography to exclude other causes of colic. When renal colic was the most likely presentation, the urologist in charge was consulted. Patients’ evaluation at this stage included non-contrast CT (NCCT) and laboratory tests (urine analysis, full blood count, and serum creatinine). The study included only patients with ureteric stones confirmed by NCCT. Patients with solitary kidney, complications secondary to obstruction (such as intractable vomiting, fever or sepsis), bilateral ureteric stones, Stage ≥3 chronic kidney disease (CKD) or those who underwent treatment of urolithiasis within the past 6 months, were excluded.

The protocol for treatment of ARC in our hospital is to administer analgesics (paracetamol, ketorolac or morphine) according to the severity of pain and patients’ comorbidities. If pain could be controlled with analgesia, the patient was referred to the urology outpatient department for planning further treatment according to major international guidelines. EI was performed in cases with persistent pain despite analgesics or recurrent pain leading to repeated ED visits. EIs were performed within 6–12 h after hospital admission. Ureteric stenting was the main intervention, while emergency URS was performed in some cases depending on the judgement of the treating urologist.

## Statistical analysis

The data of the first 200 patients were used for development of the ACR score. Univariate (chi-square test and *t*-test) and multivariate (logistic regression) analyses were performed to detect independent significant risk factors for EI. The cut-off value of each continuous significant factor was determined with receiver operator characteristic (ROC) curve analysis, and then was utilised to develop the ACR score. Sensitivity and specificity of the score were calculated using the ROC curve. The data of the next 182 patients were used for validation of the score by Spearman correlation test using calibration plots with bias-corrected calibration performed with bootstrapping (200 repetitions).

## Results

From May 2017 to April 2019, the urologists in charge were consulted for evaluation of 553 consecutive patients with ACR as the most possible diagnosis. After testing them for eligibility with study inclusion and exclusion criteria, 382 patients were included.

Of the first 200 patients, EI was needed in 119 (59.5%) because of persistent pain in 46 and pain recurrence leading to repeated ED visits in 73. EI included ureteric stents in 92 patients, emergency URS in 25, and percutaneous nephrostomy in two (after failed trial of ureteric stent). Postoperative urine cultures were infected in 16 patients. Complications of EI were observed in six patients (5%). Intraoperative ureteric perforation was reported in one case during URS and needed ureteric stenting for 4 weeks. Postoperative fever (>38.5°C) occurred in five patients and was controlled with culture-specific antibiotics (Grade 2 Clavien–Dindo Classification).

**Figure 1.**
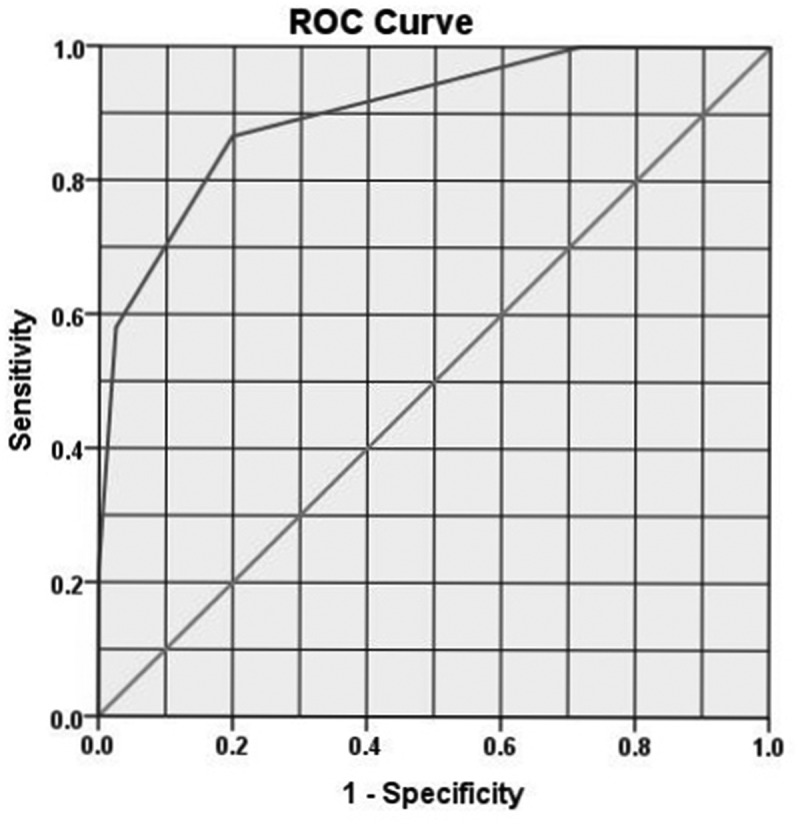
ROC curve for ARC score for predicting EI in patients with ureteric stones

Table 1 summarises the univariate analysis of risk factors for the need of EI. Significant factors on multivariate analysis were upper ureteric stone location (relative risk [RR] 3.34, 95% CI 1.16–10.16; *P* = 0.026), creatinine level (RR 1.04, 95% CI 1.02–1.05; *P* < 0.001), leucocyte count (RR 1.69, 95% CI 1.41–2.03; *P*< 0.001), and stone length (RR 1.85, 95% CI 1.44–2.37; *P* < 0.001). The cut-off values were >115 μmol/L (1.3 mg/dL) for creatinine level, >11000/μL for leukocyte count, and >5 mm for stone length. A score using these four variables was developed (Table 2). Each patient got a score from 4 to 8. The area under the ROC curve (AUC) was 0.902 (95% CI 0.862–0.943, *P* < 0.001). Score sensitivity was 86%, and specificity was 80% (Figure 1).

**Table 1.** Univariate analysis of risk factors for EI in ARC secondary to ureteric stones

Variable	No EI 81 patients	EI 119 patients	*P*
Continuous factors, mean (SD)			^#^
Age, years	41.3 (10.5)	46.2 (10.6)	0.001^#^
VAS pain score	7.1 (2.1)	7.5 (1.8)	0.108^#^
Heart rate, beats/min	80 (11)	82 (12.6)	0.210^#^
Creatinine, µmol/L	108.4 (30.7)	132.3 (40.8)	<0.001^#^
Leucocyte count, ×1000/μL	9.9 (2.9)	13.1 (3.2)	<0.001^#^
Stone length, mm	4.3 (1.6)	7.2 (3.6)	<0.001^#^
Categorical factors, *n* (%)			
Gender: Male Female	63 (39.6) 18 (44)	96 (60.4) 23 (56)	0.619^&^
Side: Right Left	40 (44) 41 (37.6)	51 (56) 68 (62.4)	0.363^&^
Stone level: Lower Ureter Upper Ureter	69 (52.3) 12 (17.6)	63 (47.7) 56 (82.4)	<0.001^&^
Hydronephrosis: Absent Present	9 (69.2) 72 (38.5)	4 (30.8) 115 (61.5)	0.029^&^
Perinephric Stranding: Absent Present	32 (61.5) 49 (33)	20 (38.5) 99 (67)	<0.001^&^

EI: emergency intervention

^#^Independent sample *t*-test

^&^Chi-square test

**Table 2.** ARC scoring system

Item	Description	Score
Serum creatinine	≤115 µmol/L (1.3 mg/dL) >115 µmol/L	1 2
Leucocyte count	≤11000/μL >11000/μL	1 2
Stone length (largest diameter)	≤5 mm >5 mm	1 2
Stone level	Lower ureter Upper ureter (proximal to upper border of sacroiliac joint)	1 2

Follow-up for patients who did not require EI was available for 70 of the 81 patients for 4 weeks. After MET (α-blockers), stone passage was documented in 54 patients (77%), five (7%) underwent elective SWL and 11 (16%) underwent elective URS.

**Figure 2.**
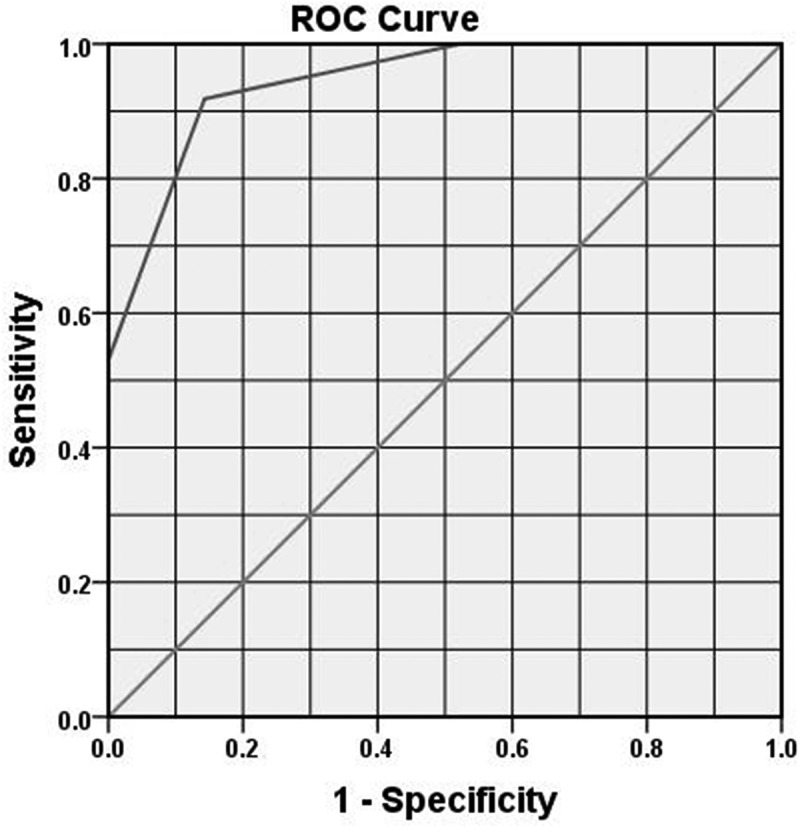
ROC curve for validation of ARC score

Of the last 182 patients, EI was needed 98 (53.8%). Validation of the score showed a strong correlation between the score and need for EI (*r* = 0.788, *P*< 0.001, bootstrapping 95% CI 0.712–0.846). The ROC curve for these patients (Figure 2) showed that the cut-off score value for the need of EI is 6, with 91% sensitivity and 86% specificity, and an AUC of 0.945 (95% CI 0.903–0.987, *P* < 0.001).

## Discussion

The incidence of urolithiasis is increasing worldwide [8]. This has been accompanied by a steady rise in the number of renal colic presentations to EDs [2], with a subsequent increase in costs for diagnosis, initial management, repeated ED visits, and treatment of the stone or complications resulting from upper tract obstruction [9]. Renal colic is one of the most severe pains that can be experienced by a patient. The goal of the treating physician is to alleviate this pain by either medications or surgical intervention. Therefore, a proper decision for the need of EI is required to relieve pain that is not responding to analgesics or to avoid repeated ED visits for management of recurrent pain.

The ARC score was developed from independent significant risk factors on multivariate analysis. Then, the cut-off value with the maximum sensitivity and specificity for each numerical component was estimated using the ROC curve. The overall score of the 200 tested patients proved to be highly sensitive (86%) and specific (80%). The second step was internal validation of the score in another 182 patients using the same test applied in internal validation of the S.T.O.N.E. [stone size (S), tract length (T), obstruction (O), number of involved calyces (N), and essence or stone density (E)] scoring system for renal stones [10]. The statistical analysis of the ARC score in the validation group (182 patients) showed a strong correlation between the score and need for EI (*r* = 0.788). This highly sensitive validated score provides the treating doctor; whether an ED physician or urologist with an objective way of identifying patients who need EI (with scores of ≥6). In addition, patients’ counselling can be based on objective parameters not only subjective pain improvement.

Another advantage of the ARC score is that it helps identify who is in need of EI during ED evaluation. Unlike previous studies that determined risk factors for intervention after discharge from ED. Papa et al. [5] in 2005 proposed a model for the need of urological intervention [SWL, URS, percutaneous nephrolithotomy (PCNL) or open surgery] within 28 days after an ED visit. They found that size of calculus ≥6  mm, pain visual analogue scale (VAS) of ≥2 and stone location above the mid-ureter were significant risk factors. The main drawback was that the authors did not internally validate this model; and Dean et al. [6] in 2016 failed to externally validate it. This may be attributed to implementing a low value of pain VAS (score ≥2) or missing other significant risk factors.

Massaro et al. [11] in 2017, evaluated predictors of urological intervention for CT-detected calculi causing ureteric obstruction. They had found that stone size, proximal ureteric location and severe pain (as indicated by higher opioid doses) were associated with the need for intervention within 30 days of presentation. They found that the degree of hydronephrosis had no impact on the need for urological intervention. The same was detected in the present study, as stone size and proximal ureteric stone location were also predictors of EI, while the degree of hydronephrosis was not, on multivariate analysis.

Wang et al. [12] in 2018, proposed clinical decision rules (CDR) for patients presenting to the ED with flank pain for any reason. They defined clinically important stones as ureteric stones that needed urological intervention within 30 days after their first ED visit. They found that CDR consisting of white blood cell (WBC) count >8400 cells/μL, previous history of stone, and hydronephrosis, were associated with high sensitivity (98.6%) but with very low specificity (26%). In the present study, abnormal WBC count was a significant factor but our cut-off value (11000 cells/μL) was higher than the Wang et al. [12] value and hydronephrosis was significant only in univariate analysis. It was reported by Song et al. [13] that 11% of patients with colic due to ureteric stones did not have any hydronephrosis and a 71% had only mild hydronephrosis.

Yan et al. [7] in 2015, evaluated predictors of urological intervention within 90 days from initial ED visit for ARC. They found that age >50 years, presence of urinary nitrites or leucocyte esterase in the urine analysis, stone size ≥5 mm, proximal ureteric stone, tachycardia, abnormal WBC count, and history of renal colic, were significant factors. In the present study, stone size, location and leucocytosis were also significant predictors of EI. However, age and heart rate were not significant risk factors.

A stone length >5 mm and upper ureteric stone location were significant risks for the need of intervention in most previous reports [5,7,11], as well as the present study. This can be explained by the natural history of ureteric calculi. A recent meta-analysis of 6642 patients, reported that spontaneous passage of ureteric stone was seen in 49% of upper ureteric stones vs 68% of distal ureteric stones, and in 62% of stones ≥5  mm vs 75% for stones <5 mm [14].

The need for intervention during the 4-week follow-up after discharge from the ED in the present study was 23% (7% SWL and 16% URS). This was comparable to previous studies, as Papa et al. [5] reported that 20% of patients and Dean et al. [6] reported that 33% of patients underwent urological intervention within 28 days after their ED visit. While Yan et al. [7] found that 84 of 220 patients (38%) who presented with ARC required urological intervention within 90 days of their primary ED visit. This higher rate may be attributed to the longer period of follow-up (90 days).

The inclusion criteria in the present study were strict because we aimed to develop a score for index patients (adult with confirmed ureteric stone) without complications. Patients with bilateral ureteric calculi, a stone in the ureter of a solitary kidney or complications secondary to upper tract obstruction were excluded because there are clear indications for EI among them without the help of any assessment score. Patients with recent treatment of urolithiasis were also excluded because they may present with ureteric stones secondary to the initial treatment (such as steinstrasse after SWL or slippage of residual stone fragments after PCNL or URS). Patients with known CKD Stage ≥3 were excluded because high creatinine levels among them may be a consequence of their impaired kidney function rather than obstruction. We included patients who had NCCT proof of ureteric calculi because it is the most commonly used imaging modality in evaluation of patients with acute flank pain. This is based on its excellent sensitivity and specificity for detection of ureteric calculi and its ability to detect other causes of flank pain [15,16].

High serum creatinine was a new risk factor in the present study. Despite presence of an unobstructed contralateral kidney, creatinine may increase because of decreased fluid intake or repeated administrations of nephrotoxic pain killer medications. However, application of the ARC score may be limited in certain patients such as immunocompromised patients because leucocytosis may not be evident. External validation of the ARC score is still needed to confirm widespread utility in evaluating patients in the ED.

## Conclusions

The ARC score is a validated, highly sensitive and specific novel score that determines the need for EI in patients with uncomplicated ARC secondary to ureteric stones.
